# Contextual reprogramming of CAR-T cells for treatment of HER2^+^ cancers

**DOI:** 10.1186/s12967-021-03132-6

**Published:** 2021-11-07

**Authors:** Zhifen Yang, Lingyu Li, Ahu Turkoz, Pohan Chen, Rona Harari-Steinfeld, Maggie Bobbin, Ofir Stefanson, Hana Choi, Violena Pietrobon, Bennett Alphson, Angshumala Goswami, Vitaly Balan, Alper Kearney, Dharmesh Patel, Jin Yang, Damla Inel, Veena Vinod, Alessandra Cesano, Bing Wang, Kyung-Ho Roh, Lei S. Qi, Francesco M. Marincola

**Affiliations:** 1 Refuge Biotechnologies Inc., Menlo Park, CA 94025 USA; 2 ESSA Pharma, South San Francisco, CA 94080 USA; 3 Department of Chemical and Materials Engineering, University of Alabama in Huntsville, Huntsville, AL 35899 USA; 4 Department of Bioengineering, Department of Chemical and Systems Biology, Stanford University, ChEM-H, Stanford, CA 94305 USA; 5 Present Address: Gilead/Kite, Santa Monica, CA 90404 USA

## Abstract

**Background:**

Adoptive transfer of chimeric antigen receptor (CAR)-engineered T cells combined with checkpoint inhibition may prevent T cell exhaustion and improve clinical outcomes. However, the approach is limited by cumulative costs and toxicities.

**Methods:**

To overcome this drawback, we created a CAR-T (RB-340-1) that unites in one product the two modalities: a CRISPR interference-(CRISPRi) circuit prevents programmed cell death protein 1 (PD-1) expression upon antigen-encounter. RB-340-1 is engineered to express an anti-human epidermal growth factor receptor 2 (HER2) CAR single chain variable fragment (scFv), with CD28 and CD3ζ co-stimulatory domains linked to the tobacco etch virus (TEV) protease and a single guide RNA (sgRNA) targeting the PD-1 transcription start site (TSS). A second constructs includes linker for activation of T cells (LAT) fused to nuclease-deactivated spCas9 (dCas9)-Kruppel-associated box (KRAB) via a TEV-cleavable sequence (TCS). Upon antigen encounter, the LAT-dCas9-KRAB (LdCK) complex is cleaved by TEV allowing targeting of dCas9-KRAB to the PD-1 gene TSS.

**Results:**

Here, we show that RB-340-1 consistently demonstrated higher production of homeostatic cytokines, enhanced expansion of CAR-T cells in vitro, prolonged in vivo persistence and more efficient suppression of HER2^+^ FaDu oropharyngeal cancer growth compared to the respective conventional CAR-T cell product.

**Conclusions:**

As the first application of CRISPRi toward a clinically relevant product, RB-340-1 with the conditional, non-gene editing and reversible suppression promotes CAR-T cells resilience to checkpoint inhibition, and their persistence and effectiveness against HER2-expressing cancer xenografts.

**Supplementary Information:**

The online version contains supplementary material available at 10.1186/s12967-021-03132-6.

## Introduction

Success of adoptive cell therapy (ACT) depends upon T cell persistence and engraftment [1, 2]. Activated T cells express co-inhibitory receptors including programmed cell death protein-1 (PD-1) that limit their persistence in vivo and yield inferior clinical benefit [3, 4]. Persistence can be prolonged by combining PD-1 blockade and ACT with chimeric antigen receptor (CAR)-engineered T cells [5, 6]. This strategy, however, is limited by cumulative costs and systemic toxicities. New methodologies aim for single ACT products with versatile functions including constitutive secretion of cytokines or pro-inflammatory factors by CAR-T cells [7], targeted delivery of anti-PD-1 single chain variable fragments (scFv) by CAR-T cells [8] or permanent knock out of the PD-1 gene by clustered regularly interspaced short palindromic repeats (CRISPR) technology [9]. Recently, Lynn et al. [10] proposed that overexpression of c-Jun suppresses PD-1 and other checkpoints resulting in enhanced T cell persistence and improved anti-tumor efficacy. However, these constitutive approaches lead to permanent alterations of the DNA structure and increased risk for neoplastic transformation preventable by a conditional system, where gene expression is modulated in a context-dependent manner without permanent alterations of the DNA structure.

We previously described a non-editing gene expression regulation strategy based on the nuclease de-activated CRISPR-associated (dCas9) protein, which offers a platform for RNA-guided DNA targeting [11–13]. Fusion of dCas9 to effector domains enabled efficient transcriptional repression or activation in mammalian cells, with the site of delivery determined by a co-expressed single guide RNA (sgRNA). Coupling of dCas9 to a transcriptional repressor, the Kruppel-associated box domain (KRAB) could silence expression of multiple endogenous genes. RNA-seq analysis indicated that CRISPR interference (CRISPRi)-mediated transcriptional repression is highly specific [12, 14]. Subsequently, we described a strategy that couples CRISPR-dCas9 genome regulation to natural or synthetic extracellular signals via G-protein-coupled receptors/ligand-sensing to target gene regulation in response to a spectrum of synthetic compounds, chemokines, mitogens, fatty acids, and hormones [15].

Here, we present RB-340-1, as an all-in-one T cell product that couples anti-human epidermal growth factor receptor 2 (HER2) CAR signaling to CRISPRi-mediated PD-1 gene suppression to prolong CAR-T cell persistence and enhance treatment outcomes. RB-340-1 consistently demonstrated higher production of homeostatic cytokines, enhanced expansion of CAR-T cells in vitro, prolonged in vivo persistence and more efficient suppression of HER2^+^ FaDu oropharyngeal cancer growth compared to the respective conventional CAR-T cell product.

## Results

### RB-340-1 conditionally prevents PD-1 expression upon exposure to HER2^+^ cancer lines and shows better expansion than conventional CAR-T cells in vitro

**Fig. 1:**
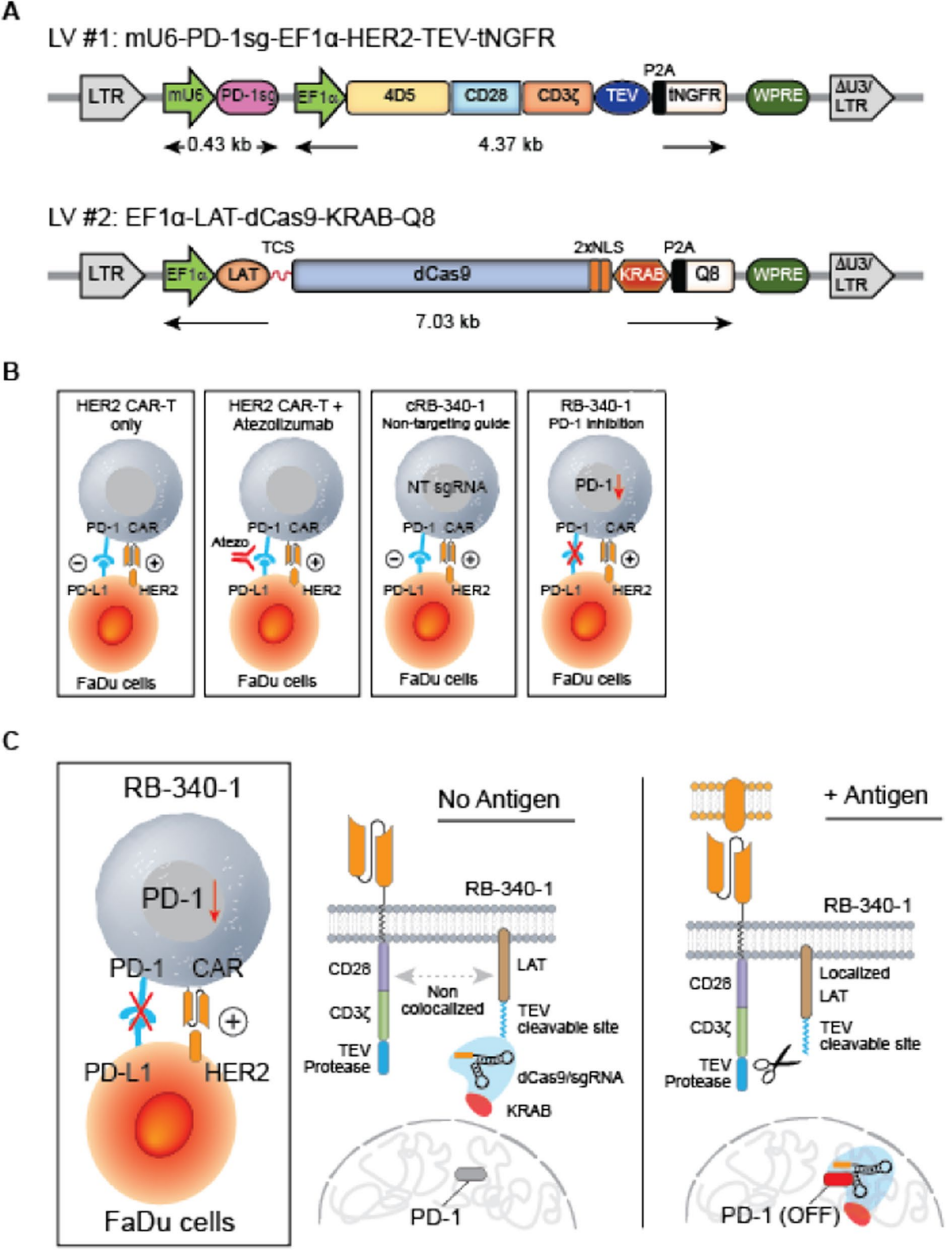
**A** Lentiviral components of RB-340-1–RB-340-1 includes two lentiviral (LV) constructs. LV#1 (HER2-TEV) encodes an anti-HER2 (4D5 clone) scFv combined to the CD28 and CD3ζ co-stimulatory domains, the TEV protease and PD-1sg targeting the TSS of the endogenous PD-1 gene. LV#2 (LdCK) encodes LAT, fused to dCas9-KRAB via a TEV-cleavable sequence (TCS). RB-340-1 contains also two extracellular tags: Q8 part of LdCK and tNGFR part of HER2-TEV. In early experiments, GFP and mCherry were used for detection of LdCK or HER2-TEV respectively. **B** Figurative representation of RB-340-1–RB-340-1 conditionally suppresses expression of PD-1 upon activation of the CAR-T cells, while respective controls such as conventional HER2 CAR-T or RB-340-1 technical control (cRB-340-1) without targeting guide are not. **C** Mechanism of activation of HER2-TEV/LdCK CRISPRi platform—activation of HER2 CAR brings TEV in proximity of LdCK releasing dCas9-KRAB for nuclear translocation to the PD-1 TSS and conditionally and reversibly suppress PD-1 expression. The figure represents the CRISPRi logic at steady-state levels of LdCK expression. However, LdCK expression varies according to the physio-metabolic status of individual cells resulting in variable degree of transcriptional activity and consequently different substrate availability for TEV cleavage. This in turn contributes to conditionality as shown in Fig. 2A

RB-340-1 includes two lentiviral constructs (Fig. 1A) [16]. The first (HER2-TEV) encodes an anti-HER2 (4D5 clone) [17] scFv combined to the CD28 and CD3ζ co-stimulatory domains, the tobacco etch virus (TEV) protease and a sgRNA targeting the transcription start site (TSS) of the endogenous PD-1 gene (PD-1sg). The second construct (LdCK) encodes linker for activation of T cells (LAT), fused to dCas9-KRAB via a TEV-cleavable sequence (TCS). Activation of CAR furthers TEV proximity to LdCK releasing dCas9-KRAB for nuclear translocation through nuclear localization sequences (NLS) and conditionally preempt PD-1 expression (Fig. 1B, C). RB-340-1 contains also extracellular tags meant to assess transduction efficiency and sort respective cell populations: truncated nerve growth factor receptor (tNGFR) is linked to HER2-TEV, and Q8, a 16-residue sequence of CD34 linked to a CD8 stalk, is part of LdCK [18]. The expression of tNGFR parallels the expression of HER2 CAR observable with anti-trastuzumab (anti 4D5 epitope-idiotype) antibody FAB95471R-100G (R&D Systems, Additional file 1: Figure S1A). Expression of Q8 is representative of LdCK expression as it follows similar kinetics (Additional file 1: Figure S1B). This indirect assessment is inevitable for dCas9 since to our knowledge no reliable intra-cellular staining method is available. In earlier pilot experiments evaluating the feasibility of the approach, green fluorescent protein (GFP) and mCherry (mChr) were also used [16]. The manufacturing workflow is shown in Additional file 1: Figure S1C.

RB-340-1 expresses LdCK conditionally. Elongation factor 1α (EF1α was selected since preliminary experiments indicated that in primary human T cells, this promoter yielded the greatest fold increase in LdCK expression following CAR stimulation. It should be noted that, although EF1α at steady states of cellular metabolism acts as a constitutive promoter, its efficiency depends upon the status of activation of the transcriptional machinery, which in turn is acutely altered when T cell transition from a dormant to an antigen-induced state of activation.

**Fig. 2:**
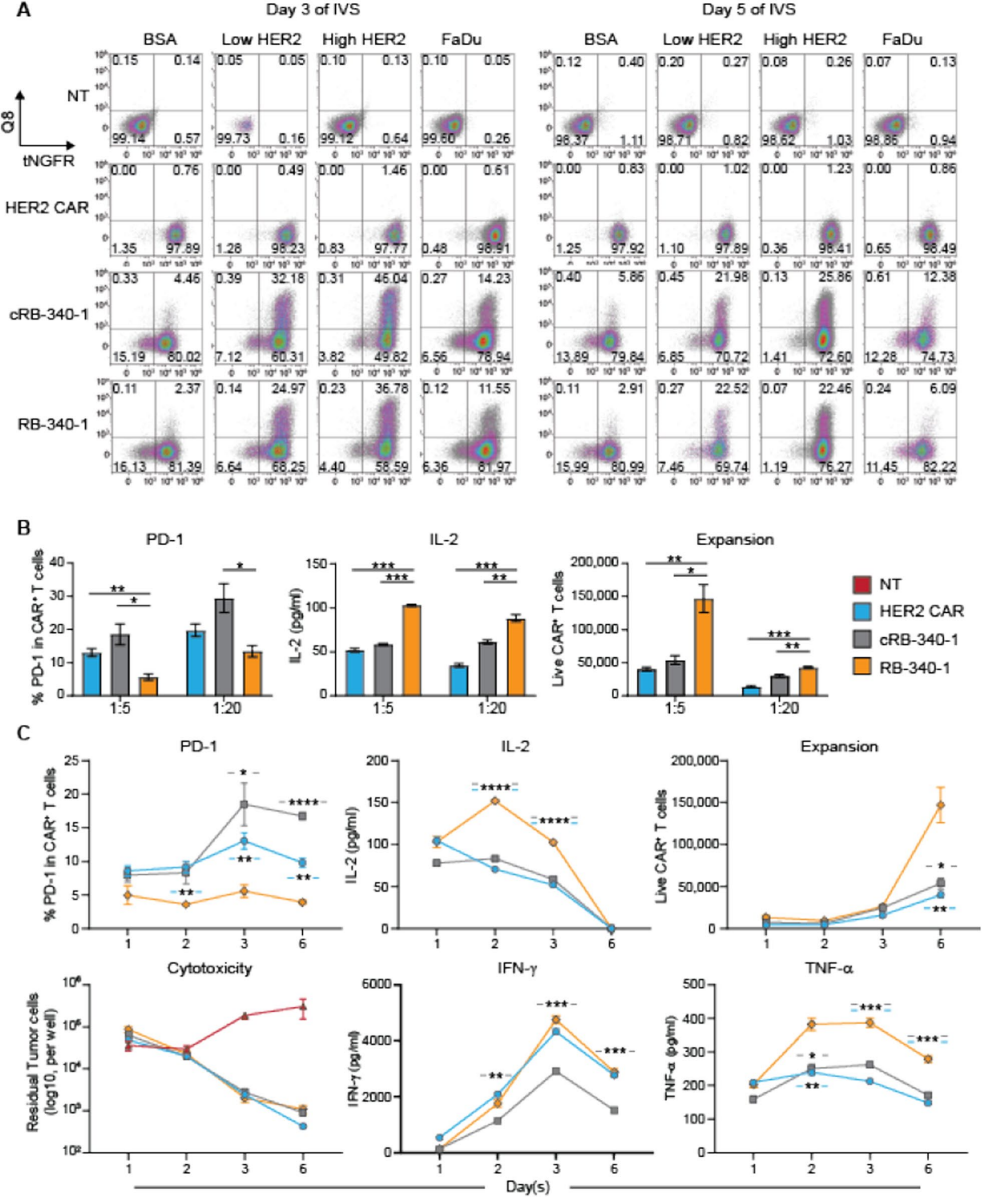
**A** Inducible expression of LdCK—Non-transduced (NT), conventional HER2 CAR, cRB-340-1 and RB-340-1 cells were stimulated at a 1:1 effector to target ratio with beads coated with bovine serum albumin (BSA), low or high densities of HER2 ectodomain (Low HER2 and High HER2) or the HER2^+^ FaDu cells and tested for expression of the HER2-TEV-linked tag tNGFR or the LdCK-linked tag Q8 at day 3 and 5 following stimulation; percentages of each cell population are displayed in the respective quadrant. **B** Modulation of cellular function by RB-340-1—PD-1 surface expression, IL-2 secretion and T cell proliferation after exposure of CAR-T cells to the FaDu cell line for 6 days at 1:5 and 1:20 E:T ratios. **C** Kinetics of RB-340-1 cellular function—representative kinetics of PD-1 expression, IL-2, IFN-γ and TNF-α secretion, cytotoxic activity and CAR-T cell expansion after stimulation with FaDu cells at 1:5 effector-to-target ratio. Similar results were obtained at 1:20 effector to target ratio (not shown). Only RB-340-1-related *p* values are ranked by the asterisks in all panels, in the lower panel the blue hyphens around the asterisks refer to statistical differences between RB-340-1 and conventional HER2 CAR and the gray between RB-340-1 and cRB-340-1. The complete statistical matrixes are shown by Additional file 6: Table S1 here and thereafter

Under the same promoter, HER2-TEV (tNGFR^+^ cells) appeared to be expressed constitutively, while LdCK (Q8^+^ cells) expression depended upon HER2-TEV stimulation (Additional file 1: Figure S1B; Fig. 2A). This discrepancy is due to the higher efficiency of transcription of the shorter HER2-TEV construct that saturates expression in baseline conditions of T cell activation. The inducibility was time-dependent peaking at day 3 from stimulation and declining afterwards. Moreover, when beads coated with the ectodomain of the HER2 protein were used to stimulate RB-340-1, we observed a density-dependent induction of LdCK. This is due to stronger stimulation through CAR/antigen engagement that could be seen also by documenting CD69 and PD-1 induction in HER2 CAR-T cells at different densities (Additional file 2: Figure S2A). Importantly, also the beads to HER2 CAR-T cells ratio determined the expression of the two activation markers highlighting the principle that overall antigen exposure and just density regulates activation.

RB-340-1 was compared against conventional HER2 CAR-T cells (HER2 CAR) bearing the construct from which RB-340-1 HER2-TEV was derived and control cRB-340-1 (identical to RB-340-1 except for PD-1sg). CAR-T cells were tested in vitro and in vivo against HER2^+^ FaDu oropharyngeal cancer cells engineered to constitutively express programmed cell death ligand-1 (PD-L1) to stabilize PD-1/PD-L1 interactions (named for simplicity FaDu hereafter). In *in vivo* studies, HER2 CAR and cRB-340-1 CAR-T cells were also administered in combination with atezolizumab (5 mg/kg intravenously twice a week).

RB-340-1 significantly preempted upregulation of PD-1 when exposed to FaDu cells compared with controls and significantly enhanced secretion of interleukin (IL)-2 and CAR-T cell expansion (Fig. 2B) the latter becoming apparent 6 days following stimulation (Fig. 2C). This is consistent with the notion that PD-1 primarily modulates proliferation/survival and prevents exhaustion [19]. The control cRB-340-1 displayed higher production of IL-2 (p value  = 0.001) and enhanced T cell expansion (p value  < 0.01) at 1:20 effector to target ratio (E:T) compared to conventional HER2 CAR (Additional file 6: Table S1). This is likely due to the overexpression of LAT peculiar to the LdCK structure, known to enhance by itself T cell activation and persistence [20]. However, these differences were slighter compared to RB-340-1. Cytotoxic activity was not significantly different between RB-340-1 and controls, IFN-γ release was superior only compared with cRB-340-1, while RB-340-1 displayed enhanced release of tumor necrosis factor (TNF)-α compared with both conventional HER2 CAR and cRB-340-1 (Fig. 2C; Additional file 6: Table S1).

RB-340-1 includes a mixture of CD4^+^ and CD8^+^ T cells collectively transduced after selection and OKT3/CD28 stimulation (Additional file 2: Figure S2B). At the time of in vivo administration ten days after transduction, the final product consistently includes a larger proportion of CD4^+^ T cells (60–70% in most cases). To investigate the functional attributes of the two populations, we compared in vitro the cytotoxic activity and survival of each component mixed at different CD4^+^/CD8^+^ ratios and exposed for three days to FaDu cells (Additional file 2: Figure S2B). Both subsets demonstrated identical cytotoxic activity. However, CD4^+^ T cells in all CAR-T cell products displayed consistently higher persistence.

As described in other systems [12, 14], CRISPRi gene targeting is specific and can be multiplexed. PD-1sg or T-cell immunoglobulin and mucin-domain containing-3 sgRNA (TIM-3sg)-containing CAR-TEV constructs specifically prevented upregulation of PD-1 and TIM-3 respectively following stimulation with FaDu cells. Only the combination of the two in a single CAR-TEV suppressed both checkpoints (Additional file 2: Figure S2C). Kinetic experiments demonstrated that PD-1 suppression by RB-340-1 was durable and specific (Additional file 2: Figure S2D).

### RB-340-1 partially suppresses the expression of checkpoints, enhances proliferation and interleukin-2 production in an exhaustion assay

**Fig. 3:**
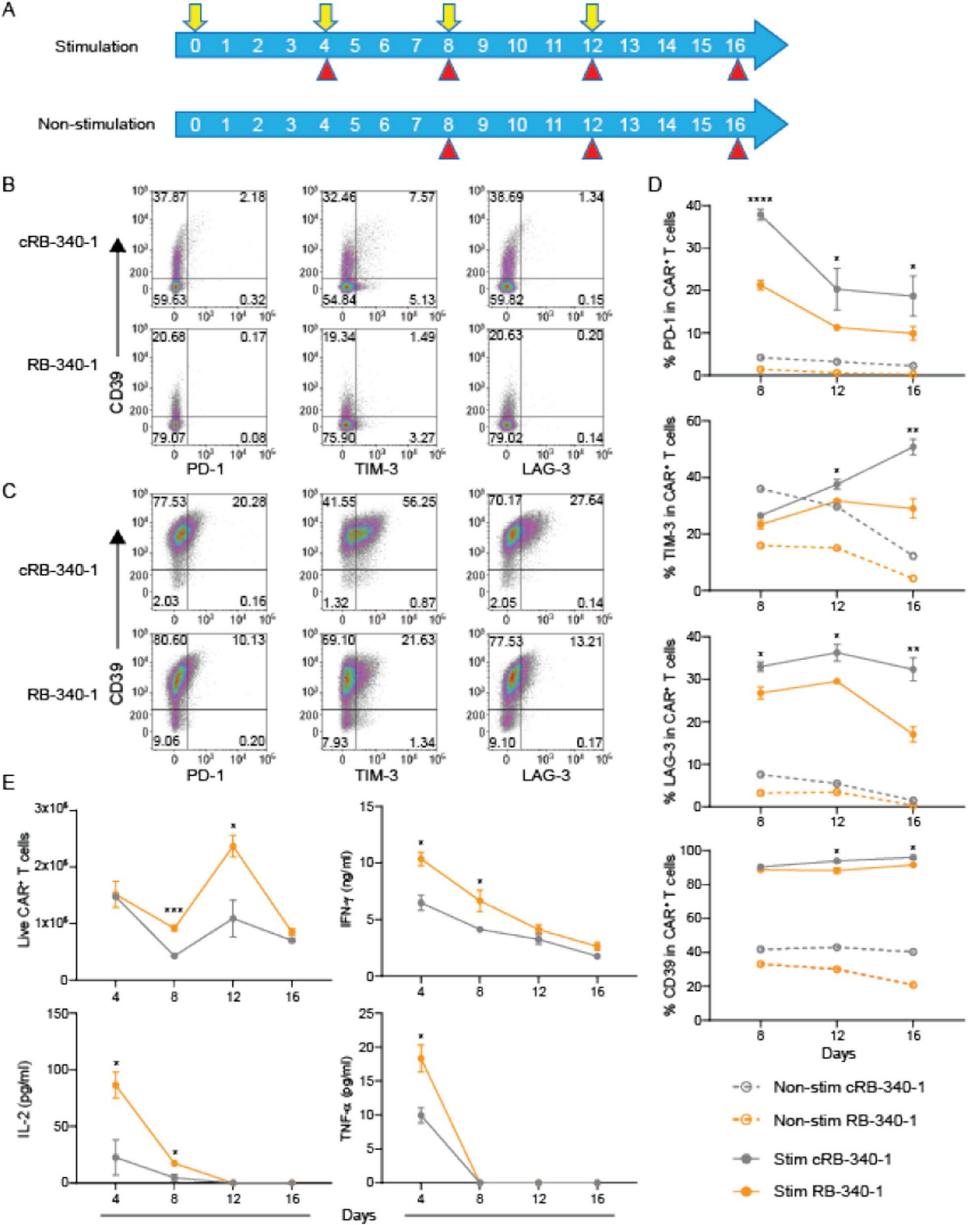
PD-1 downregulation partially mitigates CAR T cell exhaustion. **A** Schema of repetitive tumor stimulation assay. RB-340-1 or cRB-340-1 were co-cultured with FaDu tumor cells at Day 0 (E:T  = 1:4) and re-challenged with FaDu cells at day 4, 8 and 12 (indicated by yellow arrows). Non-stimulated CAR T cells were maintained in culture in the presence of 100 U ml^−1^ IL-2. At indicated time points (red arrow heads), CAR T cells were harvested and analyzed by flow. Surface expression of exhaustion markers CD39, PD-1, TIM-3 and LAG-3 at day 16 for non-stimulated (**B**) and stimulated CAR T cells (**C**). **D** Representative kinetics of PD-1, TIM-3, LAG-3 and CD39 after stimulation with FaDu cells (solid lines) and non-stimulation (dash lines). **E** Kinetics of CAR T proliferation and IL-2, IFN-γ and TNF-α secretion after stimulation with FaDu cells. *p *values are indicated by the asterisks in all graphs referring to statistical difference between RB-340-1 and cRB-340-1

RB-340-1 and cRB-340-1 cells underwent four rounds of exposure to FaDu cells at 4-day intervals (Fig. 3A) and the expression of PD-1, TIM-3 and LAG-3 was compared under baseline or stimulatory conditions (Fig. 3B, C). Under repeated exposure to FaDu stimulation, the expression of all three checkpoints was significantly prevented in RB-340-1 (Fig. 3D; Additional file 6: Table S2) suggesting that PD-1 suppression bears functional effects on T cells beyond its specific regulation. Indeed, also T cell proliferation and IL-2 secretion were enhanced during the early phases of the assay though, after repeated stimulations, the effects faded (Fig. 3E) suggesting that further improvements in T cell fitness targeting regulators of T cell differentiation/maturation could be sought in the future [10].

### RB-340-1 performs better than conventional CAR-T cells in vivo after intratumoral or systemic administration

RB-340-1 and respective controls were tested in vivo against FaDu xenograft subcutaneously-implanted in NOD-SCIC-IL2rγ^−^/^−^ (NSG) mice. Initially, 0.3 million (M) CAR-T cells per mouse were injected intratumorally to circumvent perturbations related to systemic trapping, and inefficient trafficking [21].

**Fig. 4:**
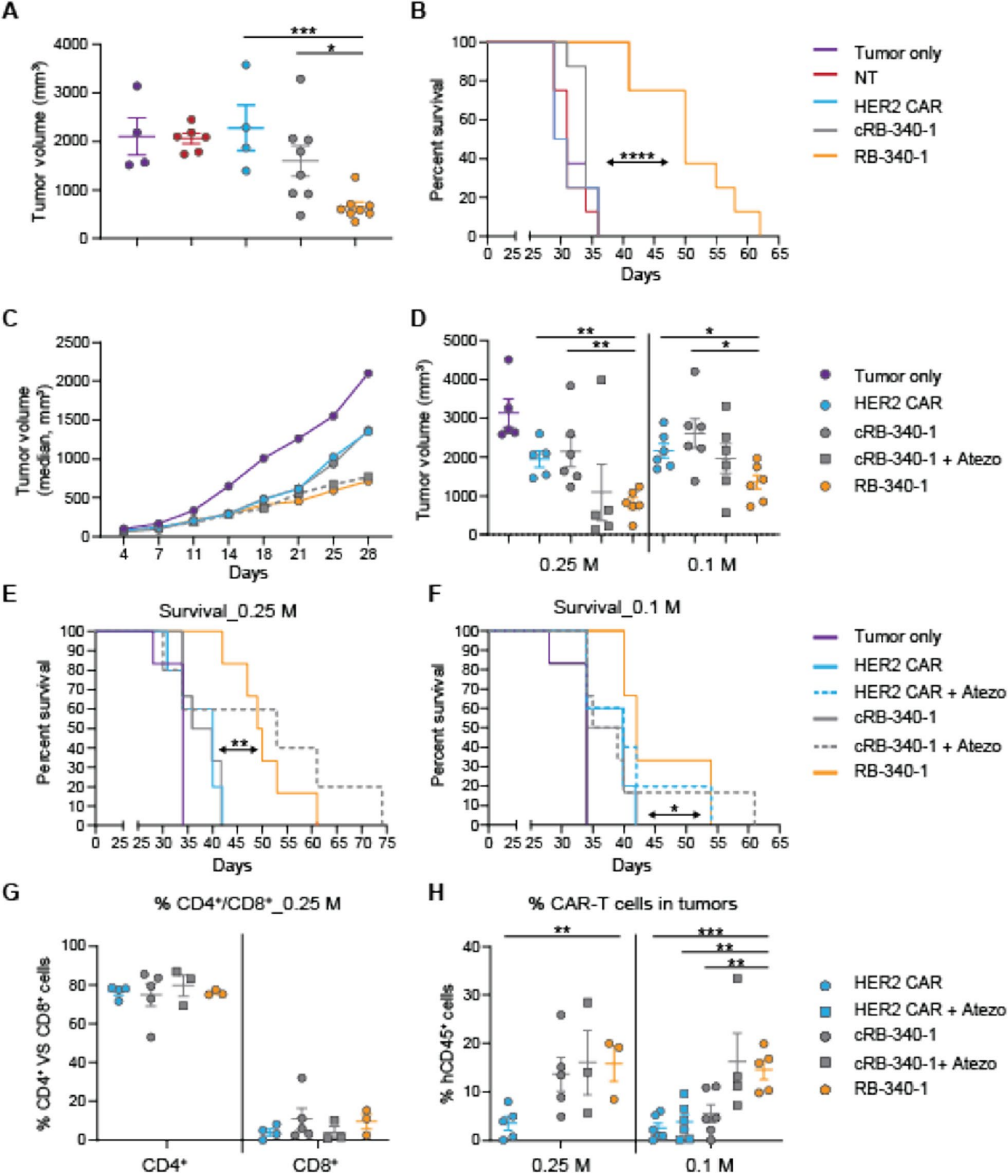
Intratumoral RB-340-1 effectiveness against FaDu xenografts—in vivo efficacy of intratumoral RB-340-1 was studied in two independent experiments with two different healthy donors. **A** In donor #1, scatter plots for tumor growth at day 30 (0.3 M CAR-T cell dose group). **C**, **D** In donor #2, average tumor growth was followed till day 28 (**C** data shown for 0.25 M CAR-T cell dose group); after day 28, mice in control groups began to die and average growth is not informative. Scatter plot comparisons were subsequently used as exemplified for day 32 for both dosage groups (**D**). RB-340-1 effect on survival—survival analysis for donor #1 (**B**) and donor #2 for both dosage groups (**E**, **F**). Intratumoral persistence of CAR-T cells—in donor #2 as an example, **G** as proportion of CD4^+^ and CD8^+^ T cells in relevant treatment groups; **H** the number of CAR-T cells colonizing the tumors was significantly higher in the case of RB-340-1 at the dose of 0.1 M cells inoculation

Compared to controls, RB-340-1 demonstrated stronger reduction of tumor growth and prolonged the survival of the mice (Fig. 4A, B; Additional file 3: Figure S3 for study design and Additional file 6: Table S3). In a subsequent experiment, two CAR-T cell doses were tested as single administrations: 0.1 or 0.25 M CAR-T cells per mouse (Additional file 4: Figure S4 for study design). RB-340-1 suppressed tumor growth more effectively than HER2 CAR or cRB-340-1 (Fig. 4C, D). Reduction of tumor growth resulted in significantly prolonged survival compared to HER2 CAR and cRB-340-1 for both the 0.25 M (Fig. 4E) and the 0.1 M doses (Fig. 4F). RB-340-1 affected tumor growth and animal survival similarly to systemically-administered atezolizumab combined with cRB-340-1 at the 0.25 M and the 0.1 M dose. Independent of treatment, residual CAR-T cells in the tumor at the time of necropsy were exclusively CD4^+^ (Fig. 4G) underlying the notion that in this immune-deficient model, CD4^+^ T cells benefit from an intrinsic survival advantage. Importantly, colonization of tumors by CAR-T cells was greater in the RB-340-1 and the cRB-340-1 plus atezolizumab groups in animals with both the 0.25 M and 0.1 M CAR-T cells (Fig. 4H).

**Fig. 5:**
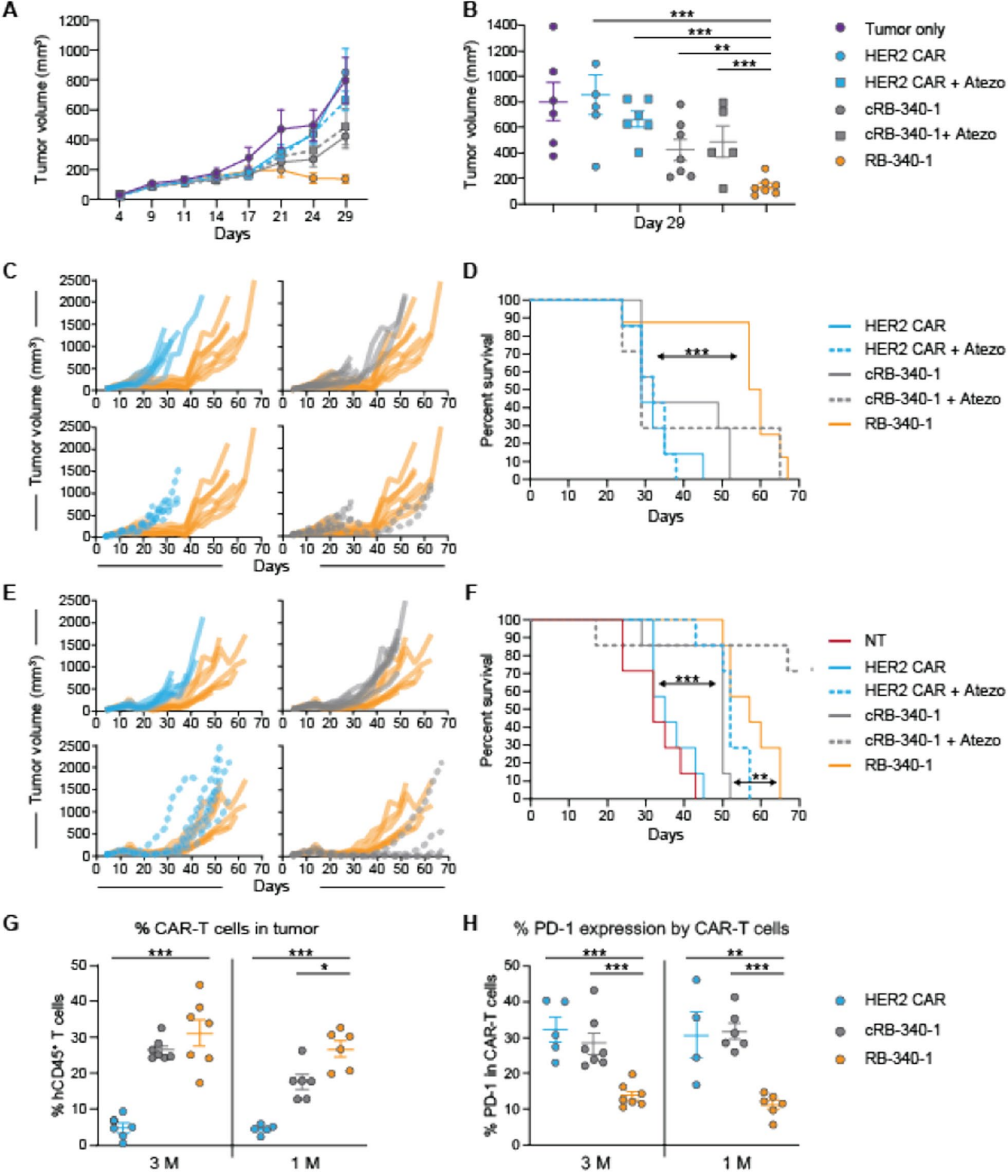
Effectiveness of RB-340-1 administered intravenously against FaDu xenografts. **A** RB-340-1 anti-tumor activity against FaDu xenografts growth—average tumor growth was followed till day 29 (data shown for 1 M dose of CAR-T cell); **B** after day 29, mice in control groups began to die and average growth is not informative. Scatter plots were subsequently used as exemplified for day 29 for the 1 M dosage group. **C** RB-340-1 effect on survival of individual mice and **D** cumulative survival for the 1 M dose; **E** RB-340-1 effect on survival of individual mice and **F** cumulative survival for the 3 M dose. **G** intratumoral persistence of hCD45^+^ CAR-T cells and **H** percentage of PD-1 expressing hCD45^+^ CAR-T cells at necropsy

Preliminary titrations suggested that a ten-fold higher number of CAR-T cells injected intravenously was needed to obtain results comparable to the intratumoral injection. Thus, 1 or 3 M CAR-T cells were administered per mouse (Additional file 5: Figure S5A, B for study design). At the 1 M dose, RB-340-1 performed significantly better than all other treatments (Fig. 5A, B). Twenty-nine days following ACT, numerous mice from the control groups began to die while survival was significantly prolonged in the RB-340-1 group compared with all the other groups (Fig. 5C, D; Additional file 6: Table S4). At the 3 M dose (Fig. 5E), results remained consistent for RB-340-1, while the other experimental groups fared better compared with their respective performance at the 1 M dose. This was particularly noticeable for HER2 CAR and cRB-340-1 in combination with atezolizumab that performed similarly to RB-340-1 at this dose but worse at the 1 M dose (Fig. 5F). As noticed for intratumoral administration, systemically treated RB-340-1 mice colonized tumors more efficiently (Fig. 5G) and maintained reduced expression of PD-1 (Fig. 5H).

### LdCK, active component of CRISPRi, is critical for effectiveness

**Fig. 6:**
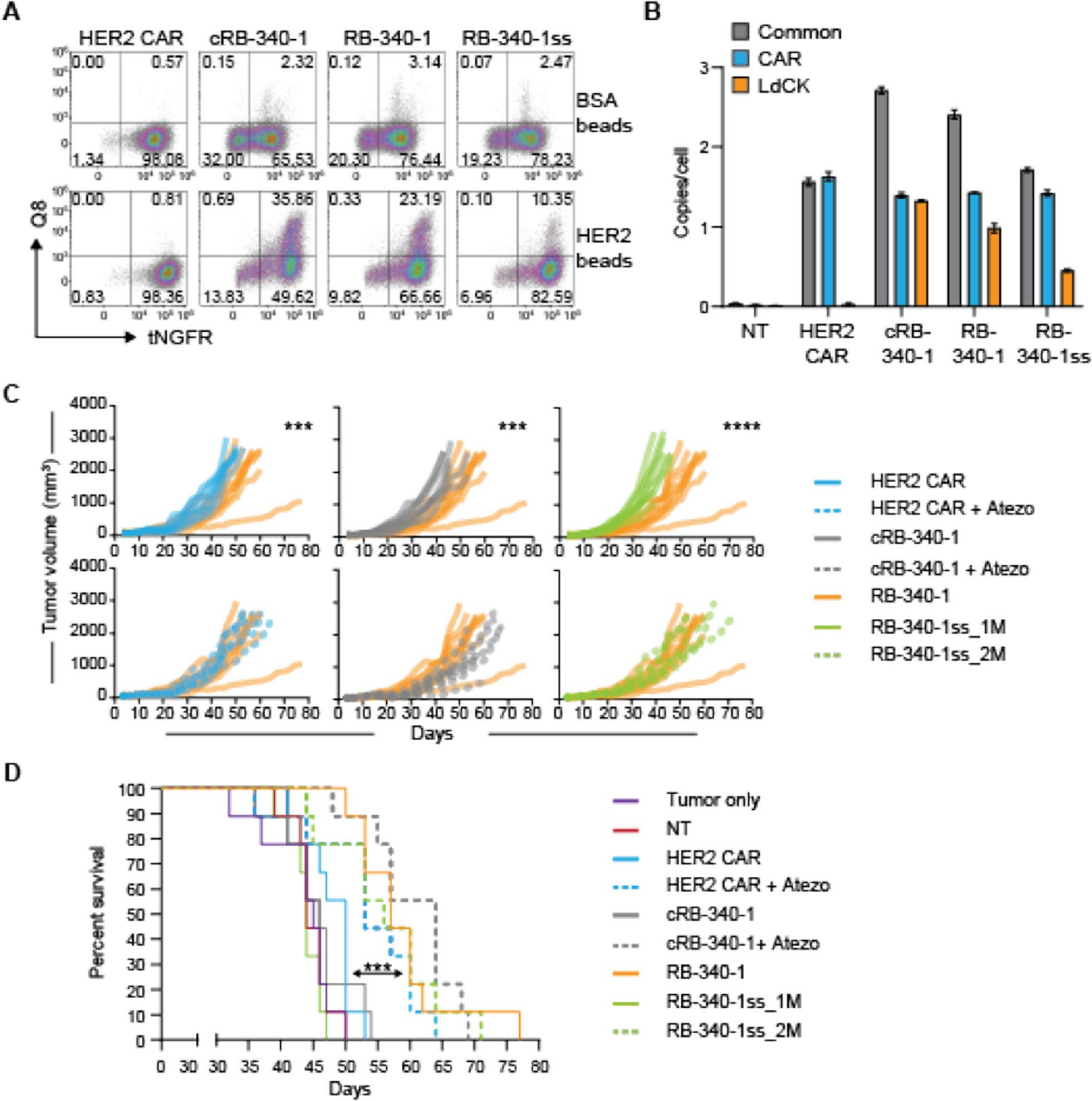
LdCK as the active element of RB-340-1 function. **A** Composition of the released product—the cells used for administration to mice were tested for tNGFR and Q8 expression. To detect LdCK presence, cells were stimulated with HER2 high beads ex vivo for three days. **B** Transduction efficiency was estimated through VCN analysis by ddPCR adopting LV-specific primers common to all constructs (gray bars) representative of the cumulative transduction rate, primers specific for the CAR-containing constructs (blue bars) and LdCK-specific primers (orange bars). **C** RB-340-1 effect on tumor growth and survival of individual mice and **D** cumulative survival (asterisks indicate significance level in survival between RB-340-1 and the other experimental groups)

With the practical purpose of addressing regulatory rationale for target product profiling, we compared RB-340-1 enriched only through tNGFR (marker for HER2-TEV) selection (Additional file 5: Figure S5A, C): RB-340-1 single-sorted (RB-340-1ss) to RB-340-1 resulting from double selection for tNGFR^+^/Q8^+^ cells (Additional file 1: Figure S1). This was done to compare the proportional contribution to anti-tumor activity of the components of CRISPRi in the same animal and assess whether the two sorting techniques would lead to comparable outcomes. RB-340-1ss were administered at the same (RB-340-1ss_1M) or at a double dose (RB-340-1ss_2M) as RB-340-1 to compensate for the lower number of double positive T cells containing both active elements. Indeed, the proportion of tNGFR^+^/Q8^+^ cells in the final product administered to the mice was approximately halved from 23.2% in RB-340-1 to 10.4% in RB-340-1ss (Fig. 6A). This proportion was extrapolated from 3 days of activation with HER2 beads to induce expression of LdCK as previously described (Fig. 2A). The transduction rate was also confirmed as vector copy number (VCN)/per cell by droplet digital PCR (ddPCR) (exemplified in Fig. 6B) using primers specific either to the LV backbone common to the two constructs (grey bars), or construct-specific markers for HER2-TEV (blue bars) or LdCK (orange bars). While the HER2-TEV marker remained consistent across RB-340-1 products, LdCK-specific VCN markers were lower in RB-340-1ss compared to both cRB-340-1 and RB-340-1. In all cases, the cumulative VCN was well below the FDA-recommended level of  ≤ 5.

RB-340-1 performed significantly better than conventional HER2 CAR and cRB-340-1 in limiting tumor growth (Fig. 6C) and prolonging animal survival (Fig. 6D; Additional file 6: Table S5, p value  < 0.001 between RB-340-1 and conventional HER2 CAR, cRB-340-1 or RB-340-1ss_1M). RB-340-1ss_2M yielded results comparable to RB-340-1 (p value  <  0.01 between RB-340-1ss_2M and conventional HER2 CAR, cRB-340-1 or RB-340-1ss_1M), while RB-340-1ss_1M fared similarly to HER2 CAR and cRB-340-1. Thus, the two sorting strategies yielded similar results as long as the number of administered double positive CAR-T cells was comparable suggesting that the active component of CRISPRi is critical for in vivo effectiveness.

**Fig. 7:**
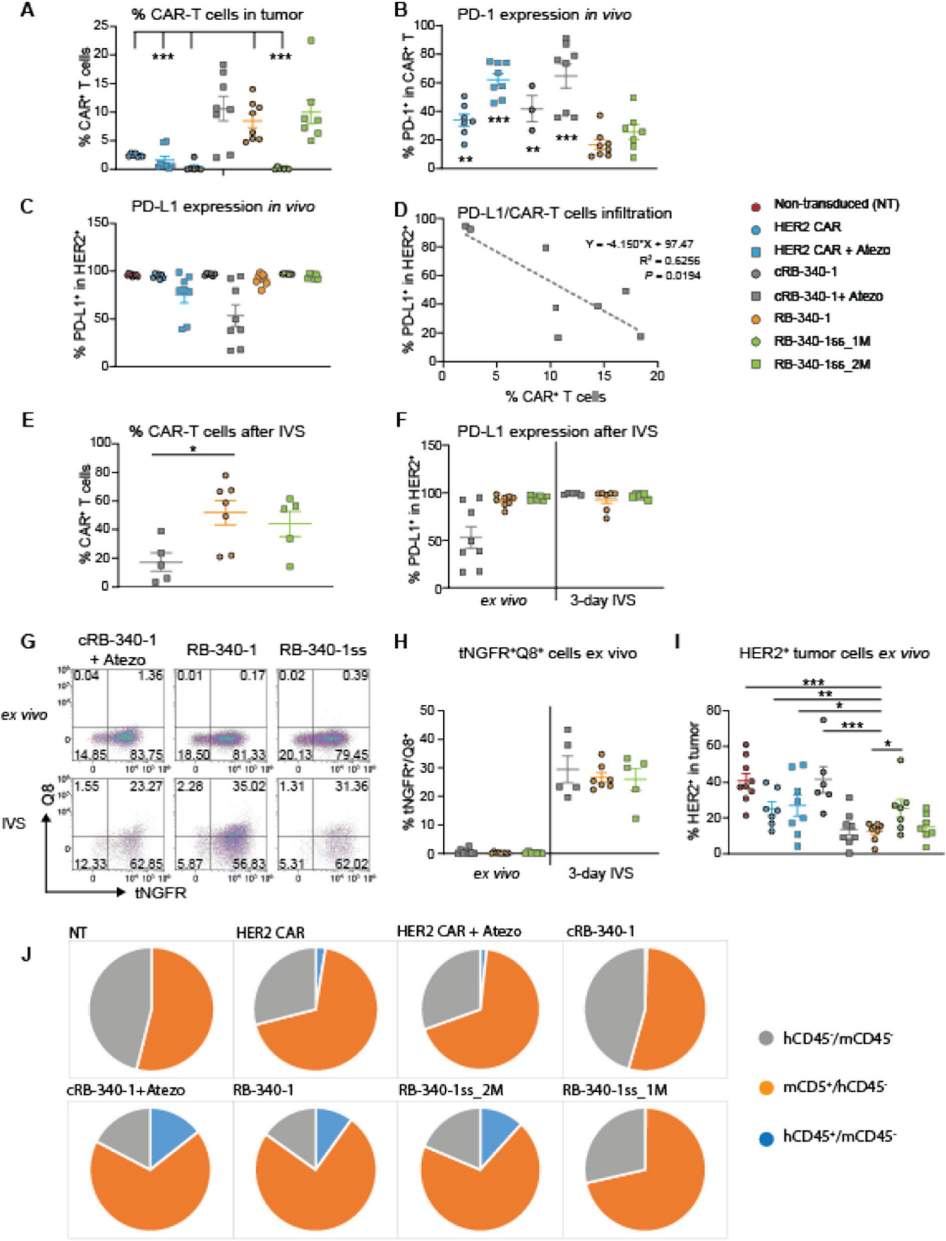
Persistence of CAR-T cells in vivo. **A** Frequency of CAR-T cells in tumors at time of necropsy for each experimental group. **B** Percent of PD-1 expressing intratumoral hCD45^+^ CAR-T cells. **C** PD-L1 occupancy by atezolizumab—ligand occupancy by atezolizumab was determined by lack of detection of PD-L1 by a second anti-PD-L1 antibody in the harvested tumors at the time of necropsy. **D** Correlation between PD-L1 occupancy and cRB-340-1 plus atezolizumab colonization of tumors. **E** percent of hCD45^+^ CAR-T cells among the mixed tumor population three days after IVS with HER2 beads in the treatment groups with sufficient number of CAR-T cells to perform functional assays as per **A**. **F** PD-L1 occupancy ex vivo and after 3 days of IVS with HER2 beads without addition of atezolizumab to the culture. **G** frequency of tNGFR^+^/Q8^+^ CAR-T cell after IVS with HER2 beads. **H** Percent of tNGFR^+^/Q8^+^ CAR-T cells ex vivo in the mixed tumor population and three days after IVS with HER2 beads. **I** percent of HER2-expressing cancer cells ex vivo at the time of necropsy. **J** tumor composition for every experimental group. Asterisks refer to comparisons between RB-340-1 and other groups displaying significant differences. The complete set of statistical values is presented in Additional file 6: Table S6

Significantly higher colonization of xenografts by CAR-T cells was observed in mice treated with RB-340-1, RB-340-1ss (2 M dose) and cRB-340-1 combined to atezolizumab (Fig. 7A; Additional file 6: Table S6). Confirming previous observations, PD-1 expression was significantly lower in RB-340-1 (Fig. 7B). PD-L1 occupancy due to PD-L1 blockade in atezolizumab-treated animals was heterogeneous among tumors in spite of the supposedly saturating doses administered (Fig. 7C) and correlated with the number of residual cRB-340-1 CAR-T cells at necropsy (Fig. 7D). This, emphasizes the critical role played by PD-1/PD-L1 blockade in this model whether attained extrinsically (cRB-340-1 plus atezolizumab) or intrinsically (RB-340-1). After three days of in vitro stimulation (IVS) of the mixed population of cells recovered from tumors with HER2 beads, only RB-340-1 and RB-340-1ss_2M expanded, while CAR-T cells from cRB-340-1-treated animals did not (Fig. 7E). This could be explained by the recovery of PD-L1 expression by cancer cells in vitro, where no atezolizumab was added to the culture (Fig. 6F). Interestingly, we could not identify in any RB-340-1-treated groups tNGFR^+^/Q8^+^ cells ex vivo. This, however, represented a contextual negativity since a high percentage of double positive cells could be recovered after three days of IVS with HER2 beads (Fig. 7G, H) suggesting that at the time of necropsy determined by rapid tumor growth, the stimulation of CAR-T cells was not sufficient to sustain the expression of LdCK. Indeed, analysis of HER2 expression by cancer cells in tumors from animals treated with RB-340-1, RB-340-1ss_2M or cRB-340-1 in combination with atezolizumab demonstrated a significantly reduced number of HER2^+^ cancer cells (Fig. 7I). This was not due to reduced expression of HER2 by individual cancer cells but by a dilution of their frequency in these groups compared with other control groups due to higher colonization of CAR-T cells and preponderance of mouse CD45^+^ cells (Fig. 7J). This dispersion indirectly allows escape of tumor cells in vivo due to reduced stochastic chances for CAR-T cell interaction with HER2 expressing cancer cells. This, in turn, limits their activation as shown in the bead titration experiment were not only the density of antigen in individual beads, but also beads to effector cell ratios regulates T cell activation (Additional file 2: Figure S2A).

All RB-340-1 containing cells remained functional in vivo since they could recover the expression of LdCK when optimally stimulated by HER2 beads ex vivo (Fig. 7G, H). Of note, cRB-340-1 CAR-T cells administered in combination with atezolizumab performed similarly to RB-340-1 monotherapy and better than conventional HER2 CAR-T cells administered in combination with checkpoint inhibition. This could be the result of a combination of factors since also cRB-340-1 experienced a selective advantage when protected by the atezolizumab combination, suggesting that the inclusion of LAT in the LdCK construct, favors T cells persistence [20] but only when other inhibitory factors such as PD-1/PD-L1 interactions are mitigated.

The better performance of RB-340-1ss_2M compared with conventional HER2 CAR, cRB-340-1 or RB-340-1ss_1M combined with the observation that the former displayed lower PD-1 levels in tumor at necropsy (Fig. 7B) suggests that the active component of RB-340-1, LdCK is quantitatively responsible for the enhanced anti-tumor effectiveness rather than the higher number of transduced cells alone.

## Discussion

The CRISPRi product RB-340-1 conditionally suppresses PD-1 expression upon exposure to HER2^+^ cancer cells resulting in better expansion in vitro and improved anti-tumor activity in vivo. In addition, at lower doses of administration, RB-340-1 can perform similarly or even better than the combination of HER2 CAR cells plus atezolizumab. This is remarkable since the atezolizumab schedule of administration adopted in this study (twice per week) far exceeds the clinically approved three-week interval [22]. Moreover, contrary to atezolizumab, the suppression of PD-1 function in RB-340-1, is limited to the delivered ACT product and it is not expected to cause any systemic effect. This is a significant improvement on current combinations considering the toxicity profile of prolonged administration of checkpoint inhibitors [23]. Moreover, the cumulative cost of checkpoint blockade and ACT combined are mitigated by this approach that comprises both mechanisms in one product. This benefit could be enhanced by the application of CRISPRi to regulate simultaneously multiple genes biologically inter-dependent such as the suppression of PD-1 and TIM-3 (Additional file 2: Figure S2C) or upregulation of IL-12, p30/p45 together with IL-15 and IL-15Ra. Moreover, conditional control of master regulators of T cell activation and differentiation such as the proto-oncogene c-Jun may overcome concerns related to their constitutive activation through a transgene that may lead to oncogenic transformation [10]. Moreover, Stadtmaurer et al. [9] sequentially deleted by CRISPR gene-editing three genes in T cells to observe frequent genome rearrangements including chromosomal translocations, which are unlikely with CRISPRi.

For the first proof of concept in humans, RB-340-1 targets relatively well-tested independent clinical entities such as HER2 and PD-1. A first-in-human phase I study could address safety, persistence and immunogenicity of dCas9-expressing CAR-T cells. Moreover, patient stratification based on moderate HER2 expression by immunogenic or immune excluded cancers, could explore effectiveness in respective cancer immune landscapes [24–26].

RB-340-1 was tailored to maximize the proficiency of each component. Inclusion of the CD28 co-stimulatory domain in CAR constructs results in constitutive basal phosphorylation of CD3 ζ-chain and enhanced CAR signaling, while 4-1BB-containing constructs recruit SHP-1 phosphatase that blunts CAR-T signaling [27, 28]. Moreover, CD28 CAR constructs interact with GRB2 leading to stronger Ca^+2^ flux and PLC-γ1 activation [29]. However, strong T cell activation, while improving effector functions in response to antigen stimulation, achieves that at the cost of premature differentiation and exhaustion [30]. We chose CD28 reasoning that constitutive activation could amplify the deployment of effector functions at early stages of tumor infiltration and trigger chemo-attraction to expand the therapeutic window, while premature exhaustion could be countered by suppression of PD-1. LAT was selected among adaptors because its stability of expression prolongs T cell activation and persistence [20]. RB-340-1 was also built to conditionally express LdCK, which contains the potentially immunogenic dCas9 of bacterial derivation, to limit its in vivo expression to pertinent conditions such as engagement with HER2-expressing cancer cells in the tumor microenvironment. Interestingly, stimulation with beads coated with different densities of the HER2 surface domain demonstrates antigen density-dependent induction of LdCK (Fig. 2A). This feature bears three benefits: (1) it enhances the conditionality of the system, (2) reduces its potential for dCas9 immunogenicity and (3) links LdCK activation to target antigen-density which in turn may widen the therapeutic index due to lower HER2 expression by benign tissues compared to cancer [31]. This is particularly relevant in view of the recent report that HER2-specific CAR-T cells can eradicate HER2^+^ tumors resistant to trastuzumab due to increased penetration within the tumor matrix and potentially lower antigen-density requirements but carry important on-target/off-target potential toward benign tissues [32].

In the current application, RB-340-1 includes CD4^+^ and CD8^+^ T cell populations, of which CD4^+^ T cells represent the larger proportion in the final product (60–70% in most cases) and enjoy prolonged persistence in immune deficient settings leading to superior anti-tumor activity as previously reported in an orthotopic glioblastoma model [33]. This may be relevant to clinical conditions where RB-340-1 will be administered in immune depleted patients.

In summary, RB-340-1 is the first application of CRISPRi toward a clinically relevant product. Although the potential of this platform extends beyond immune oncology to other immune pathologies and regenerative medicine, the current study paves the way to the first-in-human proof of concept evaluation of the safety, persistence and immunogenicity of an ortholog protein administered live in humans.

## Methods

### Lentiviral vector construction

Human codon-optimized *S. pyogenes* dCas9 was fused at the C-terminus with KRAB (Krüppel associated box) domain of Kox1 [12]. LAT-TCS-dCas9-KRAB was assembled by fusing LAT (Human cDNA, NM_001014987.2) with dCas9-KRAB-GFP (or dCas9-KRAB-Q8) and cloned into a modified pHR-SFFV lentiviral vector [34]; SFFV promoter was replaced with EF1α promoter, GZMB promoter or NFATRE promoter to get pHR-EF1αp, pHR-GZMBp and pHR-NFATREp lentiviral vectors (Fig. 1). As described previously [15], TCS sequence (ENLYFQ) was inserted in between LAT and dCas9 and was flanked by GS linker. Two nuclear export signals (NES, LALKLAGLDI and LQLPPLERLTL) flanked LAT to ensure the cytoplasmic localization of the chimera protein.

The HER2-specific scFv 4D5 sequence derives from the humanized mAb 4D5 Herceptin (trastuzumab) [17]. 4D5 scFv was amplified by PCR, based on the HER2 CAR vector purchased from Promab (PM-CAR-1024), linked to CD28 transmembrane and CD28 and CD3ζ intracellular signaling domains, and sub-cloned into pHR-EF1αp lentiviral vector. The tobacco etch virus (TEV) protease [15] was PCR amplified and cloned into the C-terminus of HER2 CAR. For HER2 CAR detection and enrichment, P2A-mCherry or P2A-tNGFR (truncated NGFR) was fused C-terminal to TEV. PD-1 sgRNA (PD-1sg) driven by mouse U6 promoter (mU6) were cloned into HER2 CAR-TEV vector upstream of the EF1α promoter for the ease of engineering the ChaCha system into human primary T cells [15].

For multiplexing experiments, PD-1sg and TIM-3 sgRNA (TIM-3sg) in tandem driven by mU6 and human U6 (hU6) promoters, respectively, were cloned into pHR-EF1αp lentiviral vector expressing HER2 CAR-TEV.

### PD-1 sgRNA design and screening

A PD-1 sgRNA library was designed based on the predicted transcription factor binding sites and nucleosome distribution among the region spanning  + / −  1 KB of the TSS of the targeted PDCD1 endogenous gene. The screening of PD-1 sgRNA was performed by transfection of 96 different PD-1 sgRNA candidates into dCas9-KRAB expressing Jurkat cells via Neon electroporation. The top 8 candidates with the most observed knockdown and fewest computationally predicted off-targets were cloned into the pLenti6 lentiviral vector for further validation in primary T cells. The top candidate PD-1 sgRNA #45 (GCTCCGCCTGAGCAGTGGAGA) was selected and cloned into pHR-EF1αp lentiviral vector expressing HER2 CAR-TEV as described above.

### Lentiviral vector production

Second-generation, self-inactivating lentiviral supernatant was produced in the 293 T packaging cell line. In brief, 70% confluent 293 T T225 flask were co-transfected with 32 µg pHR-EF1αp lentiviral vector plasmid, and 16 µg psPAX2 (Gag/Pol) and 8 µg pMD2.G (VSVG envelope) packaging plasmid DNA using 108 µl TransIT-LT1 (Mirus) and 57 µl ViralBoost Reagent (ALSTEM). The 72-h viral supernatants were harvested, filtered through 0.45 µm PVDF membrane filter unit, and layered on top of 10% sucrose solution, and followed by high-speed centrifugation at 10,000×*g* for 4 h at 4 °C. Concentrated lentiviral stocks were frozen at -80 °C for future use.

### Primary T cell isolation and CAR T cell production

Healthy donor leukopak were purchased from www.pparesearch.com. Fresh peripheral blood mononuclear cells (PBMCs) were isolated by low-density centrifugation on Lymphoprep (Stem Cell Technology) according to the manufacturer’s instructions. Pan T cells were isolated using Dynabeads Untouched Human T Cells (Invitrogen). To generate CAR-T cells, at day 0 cryopreserved Pan T cells were thawed and activated in 24-well plate coated with human anti-CD3 (OKT3, 1 µg/ml, Biolegend) and human anti-CD28 (αCD28, 1 µg/ml, BD Pharmingen™) antibodies in T cell culture medium (RPMI supplemented with 10% human serum, 2 mM GlutaMAX, 50 µM 2-Mercaptoethanol (Gibco), 100 U/ml penicillin and 100 µg/ml streptomycin (Gibco). Recombinant human IL-7 and IL-15 (Gibco) were both provided at 10 ng/ml. T cells were transduced with lentiviral vectors carrying LAT-dCas9KRAB-GFP or LAT-dCas9KRAB-Q8 at day 1 and HER2 CAR-TEV-mChr/PD-1sg or HER2 CAR-TEV-tNGFR/PD-1sg at day 2 after activation. At day 5, T cells activation was removed via transferring transduced T cells into a tissue-culture-treated 24-well plate. At day 6, double-transduced T cells were enriched through cell sorting (Sony Cell Sorter) for GFP and mCherry or Q8 and tNGFR double positive T cells. After cell sorting, T cells were maintained at 0.5 × 10^6^ − 1 × 10^6^ cells per ml in T cell culture medium with IL-7 and IL-15 (10 ng/ml). CAR-T cells were used for in vitro assays or implanted into mice at day 14 or 21 of manufacturing run.

### Cell lines

The human head and neck squamous cell carcinoma line FaDu was obtained from the ATCC (Manassas, VA). FaDu-PD-L1 (named for simplicity FaDu hereafter) was generated by transducing lentiviral vector, pLenti6.3/V5™-TOPO™ (Invitrogen), overexpressing PD-L1 (Human cDNA, NM_014143.4), followed by blasticidin selection. Cells were cultured in complete media (DMEM supplemented with 10% FBS, 100 U/ml penicillin and 100 µg/ml streptomycin (Gibco). All cells were routinely tested for potential mycoplasma contamination using the MycoAlert Mycoplasma Detection Kit (Lonza).

### Quantification of HER2.CAR and dCas9 vector copies in transduced T cells

Engineered cells were pelleted, washed with PBS and DNA was extracted using DNeasy blood and tissue kit (Qiagen). PCR amplification was performed using primers for lentiviral backbone (RRE sequence), as well as specific primers for CAR and LdCK. RPP30 amplification was used as cell number control. Droplets were generated using the QX200 droplet generator (Bio-Rad) according to the manufacturer protocol. Amplification in droplets was measured using QX200 Droplet reader (Bio-Rad) and analyzed using Quantasoft software.

### Flow cytometry

Human HER2 and PD-L1 expression on tumor cells was detected using human HER2-PE-CY7 (clone 24D2, Biolegend) and PD-L1-APC (clone MIH1, eBioscience). HER2 4D5 CAR was detected using the anti-trastuzumab idiotype Alexa Fluor 647-conjugated antibody (clone 2661E, R&D system). HER2 4D5 CAR was also detected using human recombinant HER2 protein conjugated with Alex Fluor 647. Human T cells surface phenotype and transduction efficiency were assessed using the following antibodies: NGFR-FITC (clone ME20.4, Biolegend), Q8 (clone QBEND/10, ThermoFisher), CD45-AF700 or CD45-BV605 (clone HI30, Biolegend), CD3-APC (clone SK7, Biolegend), CD4-PerCP (clone SK3, Biolegend), CD4-BB700 (clone SK3, BD Bioscience), CD8-BV510 (clone SK1, Biolegend), CD27-PE-CY7 (clone M-T271, Biolegend), CD28-BV605 (clone CD28.2, Biolegend. Expression of T cell inhibitory receptors was analyzed using PD-1-BV421 or PD-1-BV605 (clone EH12.2H7, Biolegend), TIM-3-BV605 or TIM-3-PE-CY-7 (clone F38-2E2, Biolegend), CD39 (clone A1, Biolegend), LAG-3-BV711 (clone 11C3C65, Biolegend). Live/dead discrimination was determined using LIVE/DEAD fixable Near-IR dead cell stain kit (ThermoFisher). Flow cytometry results were analyzed using Kaluza software (Beckman Coulter).

### HER2 Micro-bead preparation and HER2 beads stimulation

The ectodomain of recombinant human HER2 (Thr 23–Thr 652, Acro Biosystems) was chemically biotinylated by mixing 30 μg of HER2 protein with 20 molar excess of EZ-Link NHS-PEG4-Biotin (Thermo Scientific) in sodium bicarbonate buffer (pH 8.3). After incubation for 2 h at 4 °C on a rotator, the biotinylated HER2 was purified using Zeba desalting columns (7 kDa MWCO). For the conjugation of biotinylated HER2 onto the microbeads, 3 mg of Dynabeads of 2.8 μm in diameter coated with streptavidin (M-280 Streptavidin, ThermoFisher) were first washed twice and re-suspended in 1 ml of PBS. 3 or 15 μg of biotinylated HER2 protein was added into each microtube containing the bead suspensions, for low or high densities, respectively. Each microtube was quickly vortexed to homogenize the solution. After an overnight incubation at 4 °C on a rotator, the microbeads were washed once in PBS and further incubated with PBS supplemented with 2% (w/v) of biotinylated BSA for 1 h at 4 °C on a rotator. Finally, the microbeads were washed twice and re-suspended in PBS with 0.02% NaN_3_ for storage. The surface density of HER2 on microbeads was evaluated by flow cytometry using mouse anti-human HER2 antibody (clone 191924, R&D Systems) and QiFiKit (Agilent). The surface density values (ABC/μm^2^) were measured to be 1487 and 3628, for HER2 Low and High beads, respectively.

For HER2 beads stimulation experiments, approximately 80,000 CAR-T cells were cultured in 96-well flat bottom plates and HER2 low or high beads were added at a 1:1 beads to HER2 CAR-T cells ratio. Three days after beads stimulation, CAR-T cells were harvested and expression of HER2 CAR (tNGFR) and LdCK (Q8) were analyzed by flow cytometry.

### Co-culture experiments and cytokine production

For regular co-culture experiments, approximately 80,000 FaDu tumor cells were seeded in 48-well flat bottom plates and CAR-T cells were added 6 h later at indicated effector:target (E:T) ratios and cultured for 6 days. Triplicate wells were plated for each condition. Culture supernatants were collected and analyzed for IFN-γ, IL-2 and TNF-α by ELISA (Biolegend). Residual tumor cells, CAR-T cell proliferation and surface expression of PD-1 were analyzed by flow cytometry.

For repetitive tumor stimulation experiments, CAR-T cells were co-cultured with FaDu tumor cells at Day 0 (20,000 CAR-T cells; 80,000 FaDu cells; E:T  = 1:4) and re-stimulated with 1,00,000 FaDu cells at day 4, 8 and 12. Non-stimulated CAR-T cells were maintained in culture in the presence of 100 U ml^−1^ IL-2. At indicated time points (day 4, 8, 12 and 16), co-culture supernatants were analyzed for IFN-γ, IL-2 and TNF-α by ELISA (Biolegend). Stimulated and non-stimulated CAR-T cells and surface expression of PD-1, TIM-3, LAG-3 and CD39 were analyzed by flow cytometry.

### Animal experiments

After counting, 0.5 × 10^6^ or 1 × 10^6^ FaDu tumor cells were re-suspended in 100 µl of PBS plus matrigel (Corning) and subcutaneously injected into the right flank of 6-to-8-week-old female immune deficient NSG mice (JAX laboratory). When average tumor size reached close to 100 mm^3^, mice were randomized into different groups (see Additional files 3, 4, 5: Figures S3–5 for details), and relevant mice received anti-PD-L1 (atezolizumab, 10 mg/kg) intravenously. At the following day, for the model of intratumoral administration of CAR-T cells, a total of 0.3 × 10^6^, 0.25 × 10^6^ or 0.1 × 10^6^ of HER2 CAR-T cells, as specified in the figure legends, were injected intratumorally in a volume of 20 µl; for the model of intravenous administration of CAR-T cells, 1 × 10^6^ or 3 × 10^6^ of HER2 CAR-T cells were intravenously injected in a volume of 100 µl. In both models, mice in the atezolizumab group were continuously treated with atezolizumab (5 mg/kg) twice every week. Tumor dimensions were measured biweekly with digital calipers, and tumor volumes were calculated using the formula *V*  =  ½ (length  ×  width^2^). Mice were humanely euthanized according to IACUC protocol and tumor were resected immediately after euthanasia for further analysis.

### Isolation of tumor-infiltrating CAR-T cells

Solid tumor tissue was collected, rinsed with PBS, and mechanically dissociated using the gentleMACS dissociator (Miltenyi), and single-cell suspensions were stained with the described antibodies and analyzed by flow cytometry.

### Statistical analysis

Statistical analyses for significant differences between groups were conducted using unpaired two-tailed *t* tests using GraphPad Prism8. Survival curves were compared using the log-rank Mantel-Cox test. A *p * <  0.05 was considered statistically significant. Significance of findings was defined as: *ns*  not significant; *p  ≤  0.05; **p  ≤  0.01; ***p  ≤  0.001, ****p  ≤  0.0001.

## Supplementary Information

**Additional file 1: Figure S1.** **A** Comparative assessment of expression of HER2 CAR according to the anti-4D5 epitope idiotype antibody recognizing HER2 directly compared to the detection of the tNGFR tag. The latter, is a preferred method for sorting and phenotyping of cells since it has no direct biological effect on T cell activation/function. **B** Kinetics of expression of dCas9 and Q8 by qPCR. **C** CAR-T manufacturing process**—**CD3^+^ T cells are negatively selected from peripheral blood monocytes and stimulated with OKT3/CD28. LdCK is added for transduction a day later followed on the second day by HER2-TEV. Five days after the original stimulation the activation is removed and on the following day cells are sorted to enrich the tNGFR^+^/Q8^+^ population. The expansion is then continued till time of release between day 14 of production. Cells are also tested at that point for permanence of transduction as shown in Fig. 1A.

**Additional file 2: Figure S2.** **A** Antigen density-dependent activation of HER2 CAR**—**conventional HER2 CAR cells were stimulated with beads coated with BSA, low or high densities of HER2 ectodomain (Low HER2 and High HER2) at indicated beads to CAR-T cells ratio for expression of CD69 and PD-1 at day 3 following stimulation. **B** Cytotoxic and expansion properties of RB-340-1 cellular components**—**non-transduced (NT), conventional HER2 CAR, cRB-340-1 and RB-340-1 T cells were exposed for 3 days to FaDu cell line at 1:20 effector to target ratio. Different proportion of CD4^+^/CD8^+^ T cells were tested for cytotoxic activity (upper panel) and expansion (lower panel). The numbers in the headline refer to the proportion of CD4^+^ T cells (%) present in each group over total number of T cells. **C** RB-340-1 specificity of gene regulation**—**primary T cells transduced with LdCK plus HER2-TEV constructs including either PD-1sg, TIM-3sg or both were tested for expression of the respective target genes 5 days after stimulation with FaDu cells to induce the expression of the two checkpoints. **D** Kinetics of gene-expression regulation by RB-340-1**—**RB-340-1 (red line) and cRB-340-1 (black line) were stimulated with HER2-coated (filled shapes) or BSA-coated (empty shapes) beads and the expression of PD-1, TIM-3 and CD69 was followed in CD8^+^ T cells at baseline and 48 and 72 hours after stimulation.

**Additional file 3: Figure S3.** **A** Intratumoral administration model and study design. **B** Experimental set up**—**all treatment groups included eight mice receiving subcutaneous implantation of 0.5 million FaDu cells followed by adoptive transfer of CAR-T cells at day 10.

**Additional file 4: Figure S4.** **A** Intratumoral administration model and study design. **B** Experimental set up**—**all treatment groups included 5 or 6 mice receiving subcutaneous implantation of 1 million FaDu cells followed by adoptive transfer of CAR-T cells at day 9. Atezolizumab (10 mg/kg) was administered intravenously in the relevant groups the day before adoptive transfer (day 8) and subsequently dosed at 5 mg/kg twice a week.

**Additional file 5: Figure S5. A** Systemic administration model and study design. **B**, **C** Experimental set up**—**all treatment groups included seven mice (in **B** for Fig. 5) or nine mice (in **C** for Fig. 6) receiving subcutaneous implantation of 0.5 million FaDu cells followed by adoptive transfer of CAR-T cells at day 10. Atezolizumab (10 mg/kg) was administered intravenously in the relevant groups the day before adoptive transfer (day 9) and subsequently dosed at 5 mg/kg twice a week.

**Additional file 6: Table S1.** *P* values related to Fig. 2. **Table S2.** *P* values related to Fig. 3. **Table S3.** *P* values related to Fig. 4. **Table S4.** *P* values related to Fig. 5. **Table S5.** *P* values related to Fig. 6. **Table S6.** *P* values related to Fig. 7.

## Data Availability

All data relevant to the study are included in the article or uploaded as supplementary information. Data are available on reasonable requests.

## Abbreviations

ACT: Adoptive cell therapy
BSA: Bovine serum albumin
CAR: Chimeric antigen receptor
CRISPR: Clustered regularly interspaced short palindromic repeats
CRISPRi: CRISPR interference
dCas9: Nuclease-deactivated Cas9
ΔU3: Deleted U3 region
ddPCR: Droplet digital PCR
EF1α: Human elongation factor-1 alpha promoter
E:T: Effector-to-target ratio
GFP: Green fluorescent protein
HER2: Human epidermal growth factor receptor 2
HER2 CAR: Conventional anti-HER2 CAR-T cells
IL: Interleukin
IVS: In vitro stimulation
KRAB: Kruppel-associated box
LAT: Linker for activation of T cells
LdCK: LAT-dCas9-KRAB complex
LTR: Long terminal repeat
LV: Lentiviral vector
mChr: mCherry
mU6: Mouse U6 promoter
NLS: Nuclear localization sequence
NSG: NOD-SCID-IL2rγ^−/−^ mice
NT: Non-transduced T cells
PD-1: Programmed cell death protein 1
PD-1sg: PD-1-targeting sgRNA
PD-L1: Programmed death ligand-1
Q8: 16-Residue truncated CD34 extra-cellular domain
scFv: Single chain variable fragment
sgRNA: Single guide RNA
TCS: TEV cleavable sequence
TEV: Tobacco etch virus
tNGFR: Truncated nerve growth factor receptor
TNF: Tumor necrosis factor
TSS: Transcription start site
VCN: Vector copy number
WPRE: Woodchuck hepatitis virus posttranscriptional regulatory element
