# Supplementary material for: Contextual reprogramming of CAR-T cells for treatment of HER2+ cancers
Source: J Transl Med. 2021 Nov 7;19:459. doi: 10.1186/s12967-021-03132-6 (PMC8573881; doi:10.1186/s12967-021-03132-6)
Supplement: Supplementary file 1 — Additional file 1: Figure S1. A Comparative assessment of expression of HER2 CAR according to the anti-4D5 epitope idiotype antibody recognizing HER2 directly compared to the detection of the tNGFR tag. The latter, is a preferred method for sorting and phenotyping of cells since it has no direct biological effect on T cell activation/function. B Kinetics of expression of dCas9 and Q8 by qPCR. C CAR-T manufacturing process—CD3+ T cells are negatively selected from peripheral blood monocytes and stimulated with OKT3/CD28. LdCK is added for transduction a day later followed on the second day by HER2-TEV. Five days after the original stimulation the activation is removed and on the following day cells are sorted to enrich the tNGFR+/Q8+ population. The expansion is then continued till time of release between day 14 of production. Cells are also tested at that point for permanence of transduction as shown in Fig. 1A. [file 12967_2021_3132_MOESM1_ESM.pdf]

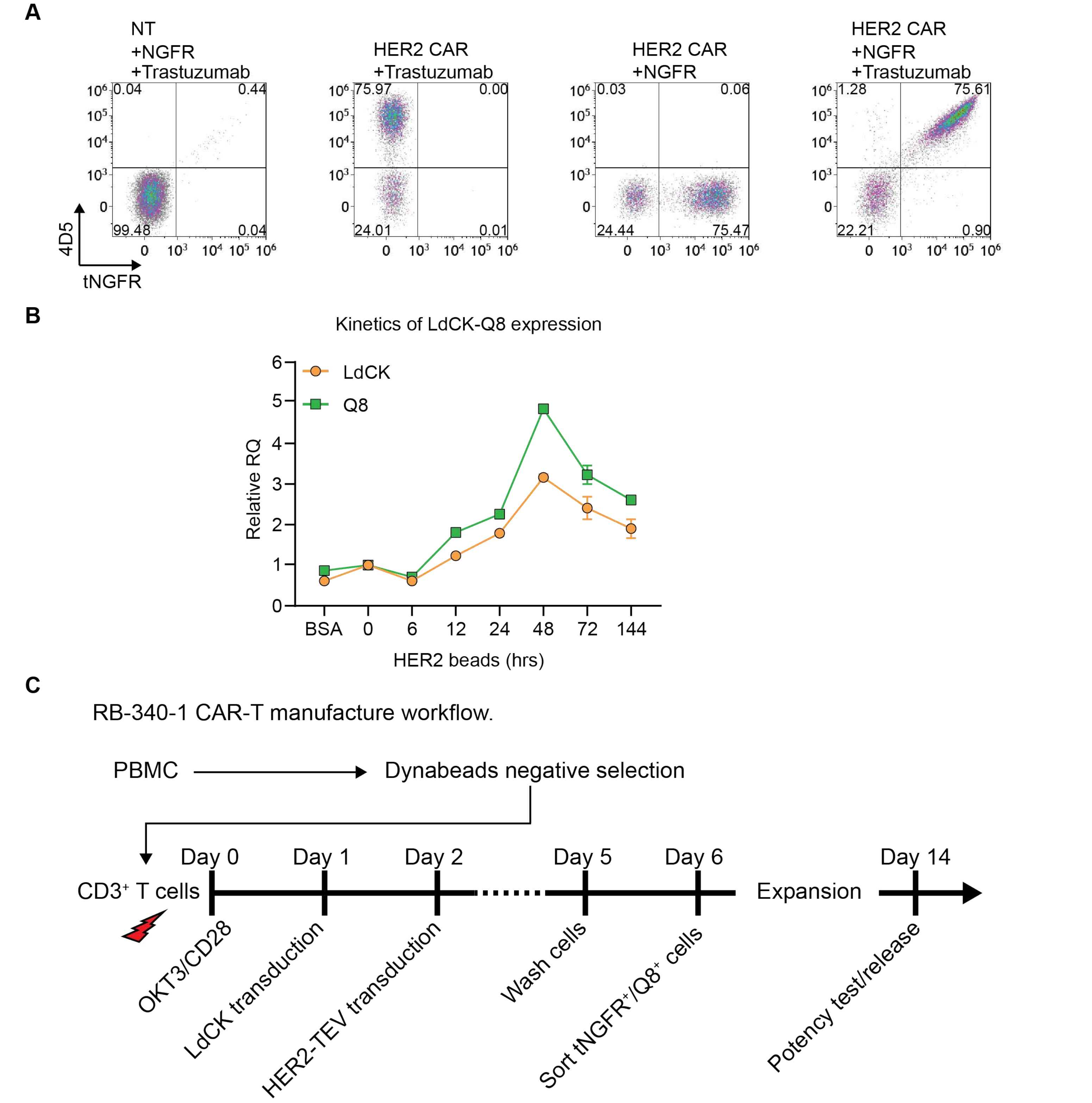

Figure S1, related to Figure 1.

(A) Comparative assessment of expression of HER2 CAR according to the anti-4D5 epitope idiotype antibody recognizing HER2 directly compared to the detection of the tNGFR tag. The latter is a preffered method for sorting and phenotyping of cells since it has no direct biological effect on T cell activation/function.

(B) Kinetics of expression of dCas9 and Q8 by qPCR. **(C) CAR-T manufacture process** – $CD3^+$ T cells are negatively selected from peripheral blood monocytes and stimulated with OKT3/CD28. LdCK is added for transduction a day later followed on the second day by HER2-TEV. Five days after the original stimulation the activation is removed and on the following day cells are sorted to enrich the $tNGFR^+/Q8^+$ population. The expansion is then continued till time of release between day 14 of production. Cells are also tested at that point for permanence of transduction as shown in ***Figure 1A***.
