# Supplementary material for: Contextual reprogramming of CAR-T cells for treatment of HER2+ cancers
Source: J Transl Med. 2021 Nov 7;19:459. doi: 10.1186/s12967-021-03132-6 (PMC8573881; doi:10.1186/s12967-021-03132-6)
Supplement: Supplementary file 2 — Additional file 2: Figure S2. A Antigen density-dependent activation of HER2 CAR—conventional HER2 CAR cells were stimulated with beads coated with BSA, low or high densities of HER2 ectodomain (Low HER2 and High HER2) at indicated beads to CAR-T cells ratio for expression of CD69 and PD-1 at day 3 following stimulation. B Cytotoxic and expansion properties of RB-340-1 cellular components—non-transduced (NT), conventional HER2 CAR, cRB-340-1 and RB-340-1 T cells were exposed for 3 days to FaDu cell line at 1:20 effector to target ratio. Different proportion of CD4+/CD8+ T cells were tested for cytotoxic activity (upper panel) and expansion (lower panel). The numbers in the headline refer to the proportion of CD4+ T cells (%) present in each group over total number of T cells. C RB-340-1 specificity of gene regulation—primary T cells transduced with LdCK plus HER2-TEV constructs including either PD-1sg, TIM-3sg or both were tested for expression of the respective target genes 5 days after stimulation with FaDu cells to induce the expression of the two checkpoints. D Kinetics of gene-expression regulation by RB-340-1—RB-340-1 (red line) and cRB-340-1 (black line) were stimulated with HER2-coated (filled shapes) or BSA-coated (empty shapes) beads and the expression of PD-1, TIM-3 and CD69 was followed in CD8+ T cells at baseline and 48 and 72 hours after stimulation. [file 12967_2021_3132_MOESM2_ESM.pdf]

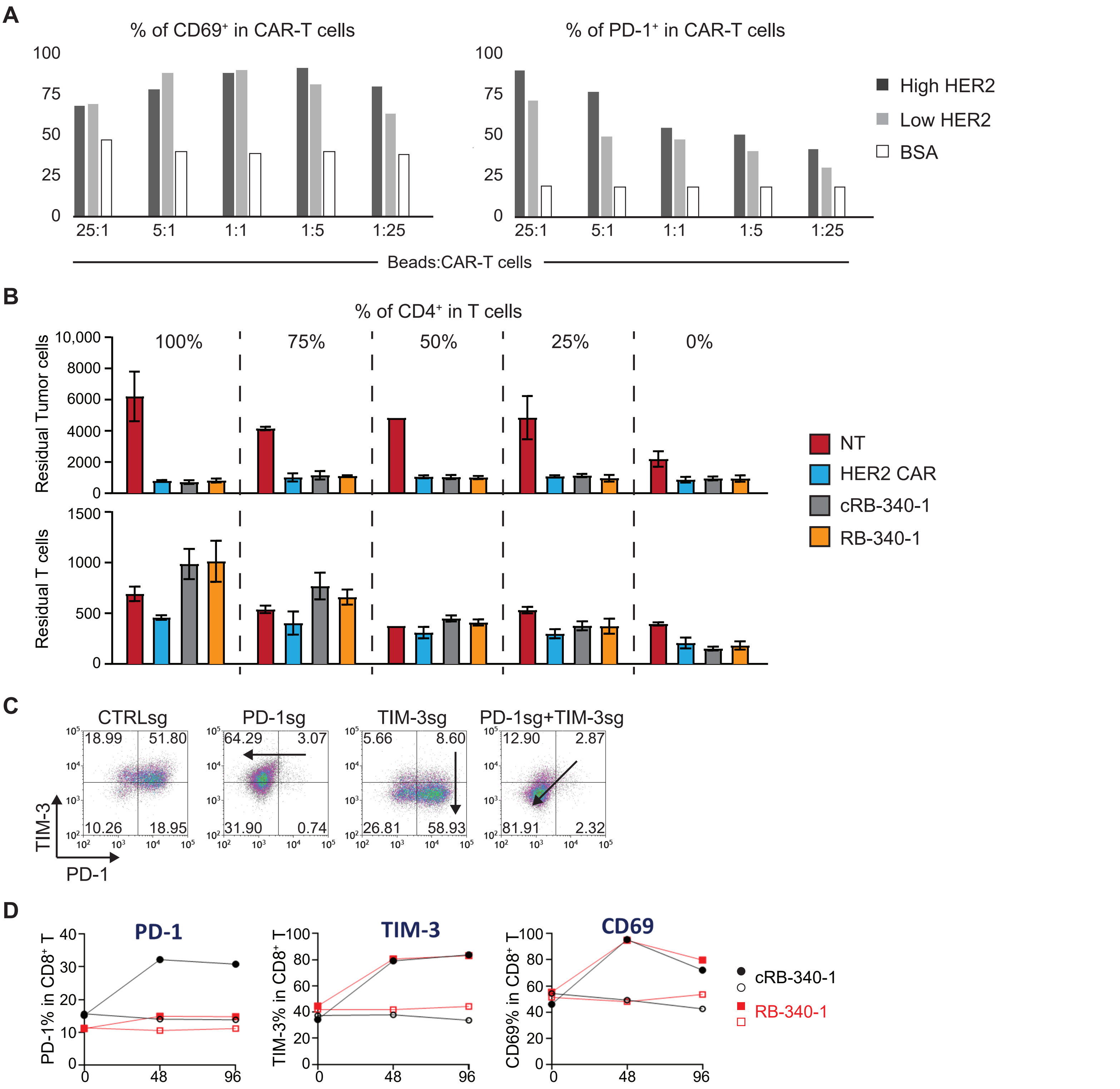

Figure S2, related to Figure 2.

(A) Antigen density-dependent activation of HER2 CAR – Conventional HER2 CAR T cells were stimulated with beads coated with BSA, low or high densities of HER2 ectodomain (Low HER2 and High HER2) at indicated beads to CAR-T cells ratio for expression of CD69 and PD-1 at day 3 following stimulation. (B) **Cytotoxic and expansion properties of RB-340-1 cellular components** – Non-transduced (NT), conventional HER2 CAR, cRB-340-1 and RB-340-1 T cells were exposed for three days to FaDu cell line at 1:20 effector to target ratio. Different proportion of $CD4^+/CD8^+$ T cells were tested for cytotoxic activity (upper panel) and expansion (lower panel). The numbers in the headline refer to the proportion of $CD4^+$ T cells (%) present in each group over total number of T cells. **(C) RB-340-1 specificity of gene regulation** – Primary T cells transduced with LdCK plus HER2-TEV constructs including either PD-1sg, TIM-3g or both were tested for expression of the respective target genes five days after stimulation with FaDu cells to induce the expression of the two checkpoints. **(D) Kinetics of gene-expression regulation by RB-340-1** – RB-340-1 (red line) and cRB-340-1 (black line) were stimulated with HER2-coated (filled shapes) or BSA-coated (empty shapes) beads and the expression of PD-1, TIM-3 and CD69 was followed in $CD8^+$ T cells at baseline and 48 and 72 hours after stimulation.
