# Supplementary material for: Contextual reprogramming of CAR-T cells for treatment of HER2+ cancers
Source: J Transl Med. 2021 Nov 7;19:459. doi: 10.1186/s12967-021-03132-6 (PMC8573881; doi:10.1186/s12967-021-03132-6)
Supplement: Supplementary file 3 — Additional file 3: Figure S3. A Intratumoral administration model and study design. B Experimental set up—all treatment groups included eight mice receiving subcutaneous implantation of 0.5 million FaDu cells followed by adoptive transfer of CAR-T cells at day 10. [file 12967_2021_3132_MOESM3_ESM.pdf]

**A** Intratumoral study design

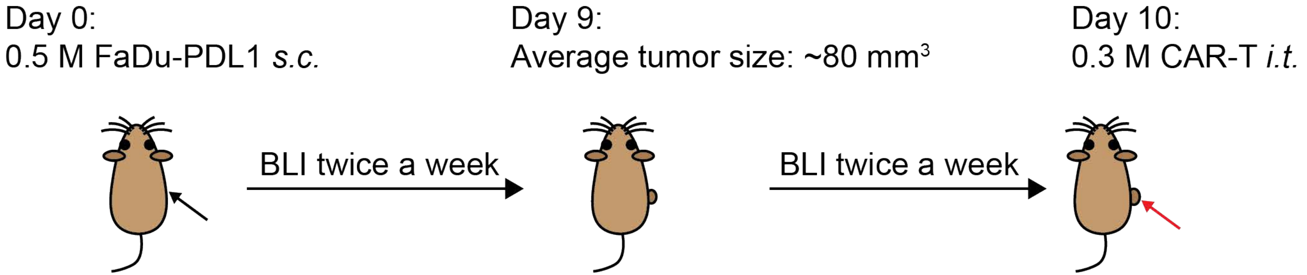


**B** Experimental cohorts

| | |
|---|---|
| PBS | n=8 |
| NT (Non-transduced T cells, 0.3 M) | n=8 |
| HER2 CAR (0.3 M CAR-T cells) | n=8 |
| cRB-340-1 (0.3 M CAR-T cells) | n=8 |
| RB-340-1 (0.3 M CAR-T cells) | n=8 |

**Figure S3, related to Figure 4A-B.**

**(A) Intratumoral administration model and study design.**

**(B) Experimental set up** – All treatment groups included eight mice receiving subcutaneous implantation of 0.5 million FaDu cells followed by adoptive transfer of CAR-T cells at day 10.
