# Supplementary material for: Contextual reprogramming of CAR-T cells for treatment of HER2+ cancers
Source: J Transl Med. 2021 Nov 7;19:459. doi: 10.1186/s12967-021-03132-6 (PMC8573881; doi:10.1186/s12967-021-03132-6)
Supplement: Supplementary file 4 — Additional file 4: Figure S4. A Intratumoral administration model and study design. B Experimental set up—all treatment groups included 5 or 6 mice receiving subcutaneous implantation of 1 million FaDu cells followed by adoptive transfer of CAR-T cells at day 9. Atezolizumab (10 mg/kg) was administered intravenously in the relevant groups the day before adoptive transfer (day 8) and subsequently dosed at 5 mg/kg twice a week. [file 12967_2021_3132_MOESM4_ESM.pdf]

A Intratumoral study design

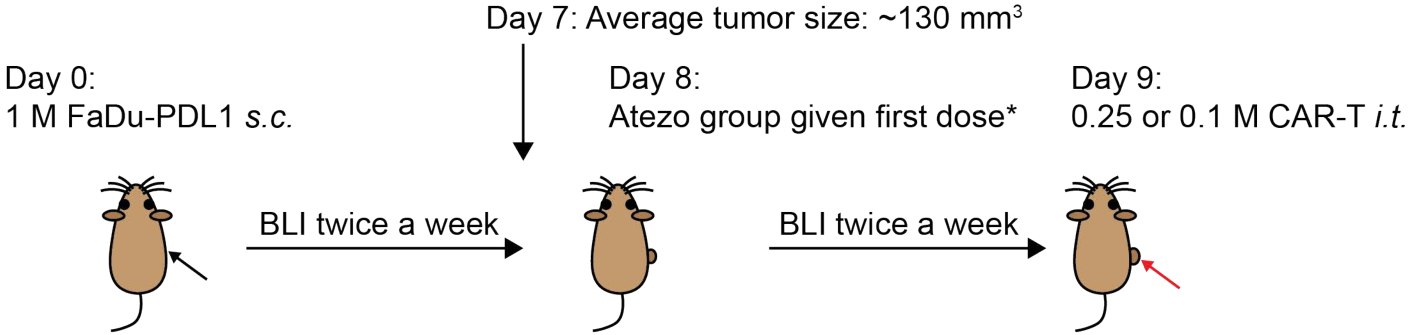

*High dose of atezolizumab by *i.v.* injection:
10 mg/kg at first dose and 5 mg/kg twice a week throughout the duration of the experiment

B Experimental cohorts

PBS	n=6
HER2 CAR (0.25 M CAR-T cells)	n=5
HER2 CAR (0.1 M CAR-T cells)	n=5
HER2 CAR plus Atezo (0.1 M CAR-T cells)	n=5
cRB-340-1 (0.25 M CAR-T cells)	n=6
cRB-340-1 plus Atezo (0.25 M CAR-T cells)	n=5
cRB-340-1 (0.1 M CAR-T cells)	n=6
cRB-340-1 plus Atezo (0.1 M CAR-T cells)	n=6
RB-340-1 (0.25 M CAR-T cells)	n=6
RB-340-1 (0.1 M CAR-T cells)	n=6

Figure S4, related to Figure 4C-H.

(A) Intratumoral administration model and study design.

(B) Experimental set up – All treatment groups included 5 or 6 mice receiving subcutaneous implantation of 1 million FaDu cells followed by adoptive transfer of CAR-T cells at day 9. Atezolizumab (10 mg/kg) was administered intravenously in the relevant groups the day before adoptive transfer (day 8) and subsequently dosed at 5 mg/kg twice a week.
