# Supplementary material for: Contextual reprogramming of CAR-T cells for treatment of HER2+ cancers
Source: J Transl Med. 2021 Nov 7;19:459. doi: 10.1186/s12967-021-03132-6 (PMC8573881; doi:10.1186/s12967-021-03132-6)
Supplement: Supplementary file 5 — Additional file 5: Figure S5. A Systemic administration model and study design. B, C Experimental set up—all treatment groups included seven mice (in B for Fig. 5) or nine mice (in C for Fig. 6) receiving subcutaneous implantation of 0.5 million FaDu cells followed by adoptive transfer of CAR-T cells at day 10. Atezolizumab (10 mg/kg) was administered intravenously in the relevant groups the day before adoptive transfer (day 9) and subsequently dosed at 5 mg/kg twice a week. [file 12967_2021_3132_MOESM5_ESM.pdf]

A

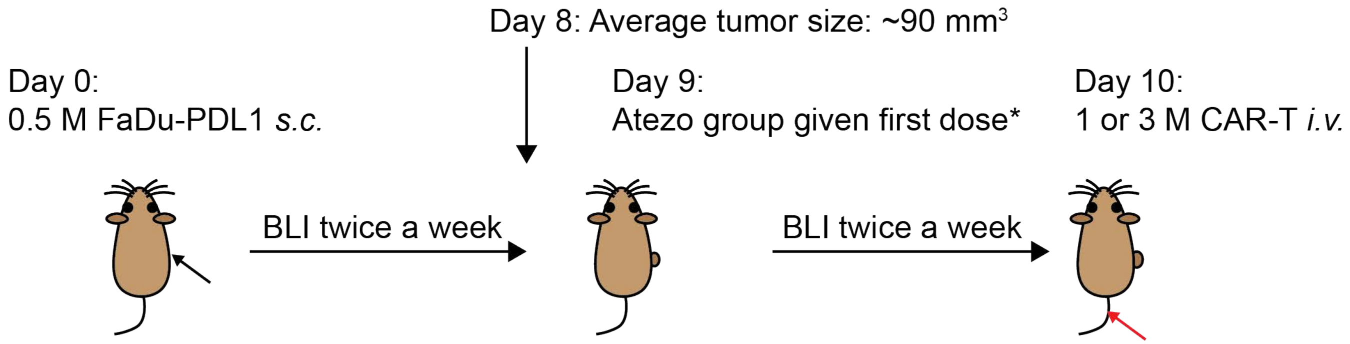

*High dose of Atezolizumab by *i.v.* injection:
10 mg/kg at first dose and 5 mg/kg twice a week throughout the duration of the experiment

B

PBS	n=7
Non-transduced T cells (3 M T cells)	n=7
Non-transduced T cells plus Atezo (3 M T cells)	n=7
HER2 CAR (3 M CAR-T cells)	n=7
HER2 CAR plus Atezo (3 M CAR-T cells)	n=7
HER2 CAR (1 M CAR-T cells)	n=7
HER2 CAR plus Atezo (1 M CAR-T cells)	n=7
cRB-340-1 (3 M CAR-T cells)	n=7
cRB-340-1 plus Atezo (3 M CAR-T cells)	n=7
cRB-340-1 (1 M CAR-T cells)	n=7
cRB-340-1 plus Atezo (1 M CAR-T cells)	n=7
RB-340-1 (3 M CAR-T cells)	n=7
RB-340-1 (1 M CAR-T cells)	n=8

C

PBS	n=9	Tumor only
Non-transduced T cells (1 M T cells)	n=9	NT
HER2 CAR (1 M CAR-T cells)	n=9	Single-sorted for tNGFR⁺
HER2 CAR plus Atezo (1 M CAR-T cells)	n=9	
cRB-340-1 (1 M CAR-T cells)	n=9	Double-sorted for tNGFR⁺/Q8⁺
cRB-340-1 plus Atezo (1 M CAR-T cells)	n=9	
RB-340-1 (1 M CAR-T cells)	n=9	Double-sorted for tNGFR⁺/Q8⁺
RB-340-1ss_2M (2 M CAR-T cells)	n=9	Single-sorted for tNGFR⁺
RB-340-1ss_1M (1 M CAR-T cells)	n=9	

Figure S5, related to Figure 5 and 6.

(A) **Systemic administration model and study design.**

(B-C) **Experimental set up** – All treatment groups included seven mice (in B, for Figure 5) or nine mice (in C, for Figure 6) receiving subcutaneous implantation of 0.5 million FaDu cells followed by adoptive transfer of CAR-T cells at day 10. Atezolizumab (10 mg/kg) was administered intravenously in the relevant groups the day before adoptive transfer (day 9) and subsequently dosed at 5 mg/kg twice a week.
